# Artificially Positive Crossmatches Not Leading to the Refusal of Kidney Donations due to the Usage of Adequate Diagnostic Tools

**DOI:** 10.1155/2013/746395

**Published:** 2013-03-28

**Authors:** G. Schlaf, B. Pollok-Kopp, E. Schabel, W. Altermann

**Affiliations:** ^1^Tissue Typing Laboratory (GHATT), University Hospital Halle/Saale, Magdeburger Straße 16, 06112 Halle, Germany; ^2^Department of Transfusion Medicine, University Hospital Göttingen, Robert-Koch-Straße 40, 37075 Göttingen, Germany

## Abstract

Allografting patients with human leukocyte antigens (HLA) which are recognized by preformed antibodies constitutes the main cause for hyper-acute or acute rejections. In order to select recipients without these donor-specific antibodies, the complement-dependent cytotoxicity crossmatch (CDC-CM) assay was developed as a standard procedure about forty years ago. The negative outcome of pretransplant crossmatching represents the most important requirement for a successful kidney graft survival. The artificially positive outcomes of CDC-based crossmatches due to the underlying disease Systemic Lupus Erythematosus (SLE), however, may lead to the unjustified refusal of adequate kidney grafts. Two prospective female recipients destined for a living as well as for a cadaver kidney donation, respectively, exhibited positive CDC-based crossmatch outcomes although for both patients no historical immunizing events were known. Furthermore, solid phase-based screening or antibody differentiation analyses never led to positive results. Immediate reruns of the CDC-based crossmatch assays using the alternative antibody monitoring system (AMS-)crossmatch ELISA resulted in unequivocally negative outcomes. Consequently both transplantations were performed without any immunological complications for the hitherto follow-up time of 25 and 28 months, respectively. We here show two case reports demonstrating an alternative methodical approach to circumvent CDC-based artefacts and point to the urgent need to substitute the CDC-based crossmatch procedure at least for special groups of patients.

## 1. Introduction

According to the transplantation guidelines of most countries or supranational societies supervising the allocation of kidneys (e.g., Eurotransplant Foundation) the existence of donor-specific anti-HLA antibodies (DSA) is regarded as a contraindication for grafting. This holds true for cadaver as well as for living kidney donations thus requiring the procedure of pretransplant crossmatching. Especially patients characterized by a previous exposure to nonself HLA antigens have (i) to be screened very carefully for anti-HLA antibodies and (ii) to carefully undergo the procedure of crossmatching with a potential kidney donor since DSA have been known for years to be associated with hyperacute or acute rejection episodes up to complete graft loss. To exclude DSA the complement-dependent cytotoxicity crossmatch assay (CDC-CM) was established in the late sixties of the last century as a standard technique by incubating the donors' lymphocytes with sera of the potential recipients in the presence of rabbit complement [1]. As a functional assay the CDC-CM detects only those antibodies which exert their detrimental allogeneic function via the activation of the complement system finally leading to the lysis of donor cells. An alternative approach was introduced with the flow cytometric crossmatch (FACS-CM) leading to the detection of both complement-activating and complement-independent DSA [2, 3]. However, both the CDC- and the FACS-CM do not lead to valid results if only cells of poor quality are available. Due to these methodological drawbacks ELISA-based crossmatch assays which are completely independent of the cell quality have been established in some tissue typing laboratories [4–6]. One of these assays, the antibody monitoring system (AMS) HLA class I/II ELISA, was implemented by us for special cases of patients not resulting in reliable and valid CDC-based crossmatch outcomes for various reasons. These false outcomes are due to the high susceptibility of the CDC-based crossmatch procedure to apparent disruptive factors which may result from underlying diseases. Based on the examples of two 41- and 43-year-old female recipients, both suffering from systemic lupus erythematosus (SLE) and awaiting a kidney donation, we present data which indicate that an implausible positive CDC-based crossmatch result should not lead to the refusal of the donation without using an alternative methodical crossmatch approach.

## 2. Case Presentations

### 2.1. Case 1: Acceptance of a Living Kidney Donation between a Mother and Her Daughter as a Consequence of AMS-ELISA-Based Crossmatching

In the first report a 41-year-old female recipient with end-stage renal insufficiency was HLA-phenotyped and genotyped for HLA-class I antigens HLA-A2; B7,57 (Bw4,6); Cw6,7 and genotyped for HLA-class II antigens HLA-DR7,15; DR51,53; DQ3(9),6. Very soon the decision was reached to perform a living kidney donation from her 60-year-old-mother typed HLA-A1,2; B8,57 (Bw4,6); Cw6,7 for class I and HLA-DR7,17; DR52,53; DQ3(9),2 for class II. Thus, the resulting mismatch scheme of the graft covering only the A-B-DR antigens, which are regarded as the most important, was determined as 1-1-1 (MM A-B-DR). Due to the compatibility of the Cw antigens no additional targets for an immune response against them existed. Concerning the codominant inheritance the degree of HLA matching between the mother as prospective donor and her daughter was as expected. In accordance with different guidelines of the particular kidney transplant centres the crossmatching procedure has to be performed two or three times prior to living kidney grafting, respectively. Any positive outcome of crossmatching, however, leads to the refusal of a living kidney donation. Donor-specific antibodies appeared first to be detectable in October 2010 using the conventional CDC-CM with peripheral blood lymphocytes, as well as isolated T- and B-lymphocytes as this procedure is dictated by the Eurotransplant Foundation and at present represents the standard method to detect alloantibodies in a prospective recipient against HLA antigens of donor cells. For the isolation of T and B cells the tetrameric antibody complex technology crosslinking unwanted cells to red blood cells and their consecutive elimination by density gradient centrifugation was used (System RosetteSep, Stem Cell Technology, Grenoble, France). After their initial isolation the cells were incubated with serum of a chosen recipient before adding complement proteins from a rabbit. In this assay the complement system is activated via the classical activation pathway only by those antibodies which have been bound to the cells in the first incubation step and which belong to the complement-system-activating IgM/IgG (sub)isotypes. The result was determined by two-colour fluorescence microscopy. Dead cells attacked by the complement system were stained red by the intercalating reagent ethidium bromide, whereas vital cells exhibited a green staining pattern due to the active uptake of acridine orange. The intensity of the complement reaction was categorized by indicating the percentage of dead (red) cells using the score system of the National Institute of Health (Washington, USA) as described in the legend of Table 1. 

**Figure 1:**
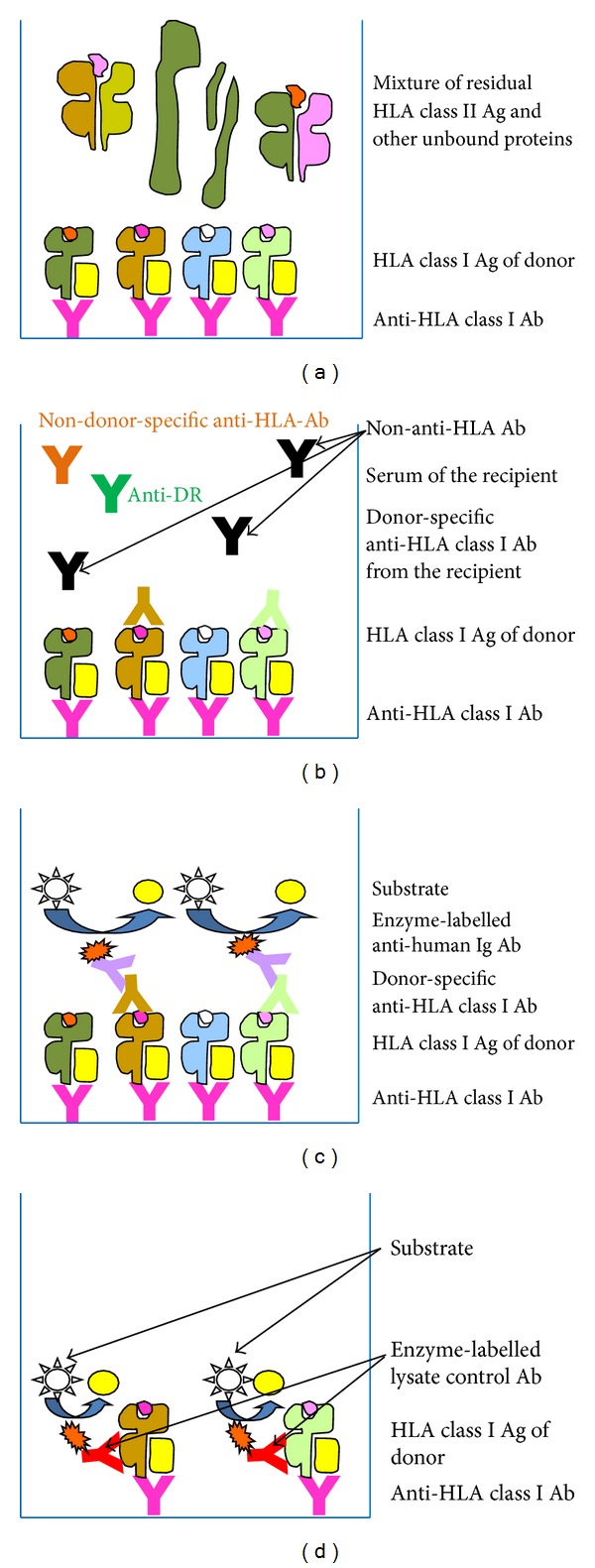
Flow diagram of the AMS-ELISA shown for the detection of HLA class I molecules. (a) Binding of the donor's solubilized HLA class I molecules by monoclonal capture antibodies recognizing a monomorphic epitope on HLA class I molecules. (b) Binding of the donor-specific anti-HLA antibodies out of the recipient's serum to the HLA molecules of the donor. (c) Binding of alkaline phosphatase-conjugated secondary antibodies to the recipient's bound donor-specific anti-HLA class I antibodies and subsequent colour reaction. The original protocol was modified by substituting the human IgG-specific by a human IgG/M/A-specific secondary antibody. (d) Lysate control using an alkaline phosphatase-conjugated monoclonal detection antibody directed against a second monomorphic epitope to confirm the immobilization of a sufficient amount of HLA molecules by the solid-phase-bound capture antibody. The AMS-ELISA variant for the identification of donor-specific antibodies directed against HLA class II molecules is correspondingly designed.

The first CDC-based crossmatch (05/2010) using peripheral blood lymphocytes (PBL) as well as isolated T and B cells was negative (Table 1(a)) in best accordance with a screening ELISA not detecting any anti-HLA antibodies (Table 1(b)). Unexpectedly the second crossmatch (10/2010), performed five months afterwards, was positive for PBL, T, and B cells without any known immunizing event in the meantime (Table 1(a)). The living kidney donation intended for the next day was cancelled. Due to the implausibility of this second CDC-based crossmatch an immediate rerun using the alternative AMS-crossmatch ELISA (GTI, Waukesha, USA; FDA number BK060038 from June 26, 2006) was performed which resulted in an unequivocally negative outcome. The workflow of this procedure is shown in Figure 1. First detergent lysate of a given donor's material was pipetted into the wells of ELISA strips precoated with capture monoclonal antibodies (mAb), directed against a monomorphic epitope on HLA class I or class II molecules, respectively (Figure 1(a)). After the first incubation the strips were washed and incubated with the recipients' sera. These sera may contain DSA to be detected in this assay since they serve as detection antibodies in the case of recognizing the immobilized HLA molecules of a given donor (Figure 1(b)). After additional washing steps the samples were incubated with secondary alkaline phosphatase-conjugated anti-human IgG antibodies to recognize the immobilized donor-specific (detection-) antibodies (Figure 1(c)). This last step was modified in our laboratory through the use of secondary antibodies directed against IgG/M/A isotypes of primary human antibodies. To validate the data three controls had to be performed. (i) The lysate controls (LCRI/II) consist of a second enzyme-labelled mAb for the detection of immobilized HLA class I or class II molecules, respectively, by recognizing a second monomorphic epitope on the immobilized HLA molecules (Figure 1(d)). This control provides evidence that the amount of the donors' HLA molecules is sufficient to get a signal. (ii) The positive control (control of reagents) consists of freeze-dried control lymphocytes and a positive serum sample directed against HLA class I and class II molecules. This control demonstrates the functionality of the reagents, even if the preparation of the donor's material is insufficient to generate a signal. (iii) The negative control corresponds exactly to the positive control of reagents with the difference that an irrelevant human serum which is negative for HLA antigens is used. The value generated by the recipient's serum under investigation has to exceed twofold the background value of the negative control to reach a positive classification. The consequent research for a potential disruptive factor falsifying the outcome of the CDC-based crossmatch led to the formerly unknown diagnosis of lupus disease by the patient's nephrologists. The third conventionally performed CDC-based assay (01/2011) after the acute lupus episode was again negative in best accordance with the AMS-ELISA carried out in parallel as a reference assay (Table 1(a)). All sera used for crossmatching were additionally analysed for the detection of anti-HLA class I and anti-HLA class II antibodies using Quik Screen and B-Screen assays (GTI Diagnostics, USA) as shown in Table 1(b), respectively. Furthermore, the sera taken in 10/2010 and 01/2011 were additionally tested using Luminex-based antibody differentiation analyses at the single donor level (Lifecodes class I/II ID, via Inno-Train, Germany) (Table 1(b)). Both Quik Screen (for anti-HLA class I) and B-Screen (for anti-HLA class II) ELISA-based screening assays were modified using secondary antibodies specific for primary antigen-recognizing antibodies of the IgG and IgM isotypes. No screening or differentiation assay resulted in detectable anti-HLA antibodies (Table 1(b)). Consequently the transplantation was performed at the following day without any immunological complication for the follow-up time of about 25 months.

### 2.2. Case 2: Refusal of an Implausible Positive CDC-Based Crossmatch Result through the Additional Use of the AMS-Crossmatch ELISA with a Negative Outcome during Emergency Duties

In the second report a 43-year-old female cadaver kidney recipient with renal endstage disease was phenotyped and genotyped for HLA class I antigens HLA-A1; B8 (Bw6); Cw7. For HLA-class II antigens she was typed HLA DR17; DR52; DQ2. After 17 months on the kidney waiting list the patient got a kidney offer which was HLA compatible at the low resolution typing level not only for the antigens A-B-DR (resulting mismatch scheme 0-0-0) but also for the HLA class I Cw and the HLA class II DQ antigens. In spite of this high HLA compatibility the CDC-based prior-to-grafting-crossmatch performed in October 2010 was positive using the current serum of the patient taken in October 2010 and a historical serum which had been taken one year ago in October 2009 (Table 2(a)). This second serum had historically been detected to be positive for anti-HLA antibodies in the CDC-based cell tray screening procedure although no distinct specificities had been identifiable. Consequently this historical serum with a panel reactivity (PRA) of 68% had been stored as positive sample (PRA > 5%) to be available for future crossmatching. The current serum taken in October 2010 was as well positive (61% PRA) in the cell tray assay but not in the solid-phase-based screening ELISA (Table 2(b)). Thus, the quarterly performed screening assays to define the degree of anti-HLA preimmunization resulted in positive outcomes only in the CDC-based cell tray analyses performed in accordance with the guidelines of the European Federation of Immunogenetics (EFI) only once a year during the autumnal screening runs (Table 2(b)). Both cell tray analyses, however, did not lead to definable antibody specificities. Additional identification assays such as the DynaChip solid phase array (Life Technologies/Invitrogen, UK) employed for three times (July 2009, October 2009, 10/2010) never resulted in detectable anti-HLA antibodies (Table 2(b)). These divergent results of solid-phase-based assays on one hand and cell tray-based antibody detection assays on the other hand prompted us to ask the patient's dialysis centre already after the first CDC-based autumnal screening (10/2009) for disruptive factors leading to the formerly unknown information that the patient suffered from lupus disease. Thus, we were prepared that the outcome of any CDC-based prior-to-grafting crossmatch would most probably be positive due to this underlying disease. In accordance with a prior agreement with the transplant centre the obligatory CDC-based pretransplantation crossmatch was performed in parallel to the alternative AMS-crossmatch ELISA (Figure 1) which led to an unequivocally negative outcome. Based on the negative result of the AMS-ELISA the transplantation was performed, and no immunological complications for the hitherto existing follow-up time of about 28 months were observable.

## 3. Discussion and Conclusions

Based on two exemplary cases it was the aim of the current paper to present data on certain situations of crossmatching which do not lead to reliable results when the conventional CDC-based technique is used. This technique was established as the prototype of crossmatching in the late sixties and unquestionably improved the outcome of transplantations since hyper-acute and acute rejections have highly been reduced after this functional assay's implementation [7, 8]. In spite of this progress the adequate allocation of organs remains a problem if it is solely/mainly based on an assay which is characterized by several drawbacks. In this context the CDC-based crossmatch assay (i) detects only antibodies of complement-activating isotypes, (ii) requires donor cells of a high degree of vitality often not available, and (iii) is obviously highly susceptible to artificially positive reactions as described by us on the basis of two current cases. Although the general phenomenon that the CDC-based crossmatch may be characterized by false-positive outcomes due to autoimmune diseases has sometimes been mentioned [9–11] the literature lacks concrete examples which demonstrate different outcomes of CDC-based and alternative solid-phase-based assays using identical serum samples as exemplarily shown in these reports. As additionally shown by the second case report, the artificially positive reactions hold true both for direct CDC-based crossmatching (Table 2(a)) using cells of a given donor and for CDC-based antibody screening using cell trays with selected panel cells as a basis for virtual crossmatching (Table 2(b)). Using both CDC-based assays without any additional solid-phase-based assay for crossmatching and for anti-HLA antibody detection/identification would presumably not lead to valid results for patients suffering from certain autoimmune diseases such as systemic lupus erythematosus. Thus, the two cases presented here suggest the use of the AMS-ELISA to overcome the general problem of artificially positive CDC-based crossmatch results. However, the results presented are not in accordance with the latest attempts to declare the CDC-based procedures as the leading method for the definition of immunized patients as proposed recently [12]. Quite in contrast they show the general insufficiency of CDC-based assays to lead to valid results under the conditions described demonstrating the urgent need to substitute them at least for special groups of patients. Whether these artificial outcomes of CDC-based assays hold true for other diseases of the immune complex type III such as rheumatoid arthritis will have to be examined.

We present two cases of CDC-based pretransplantation anti-HLA antibody diagnosis completely insufficient to correctly diagnose the existence of DSA in potential kidney allograft recipients due to SLE-based false-positive reactions. Detecting these antibodies has been known for years to be highly important for the survival of kidney allografts. In order to avoid the CDC-based crossmatch artefacts dithiothreitol/dithioerythritol (DTT/DTE) as reducing agents were early introduced to reduce the confounding influence of antibodies of the IgM isotype. Apparently this procedure led to an improvement of CDC-based crossmatch results' interpretability in many cases [13–16]. Hence in HLA diagnostics these agents are generally used to destroy antibodies of the IgM isotype with the aim to avoid the detection of autoantibodies. However, for more than ten years it has been known that autoantibodies which are generated during autoimmune-mediated diseases such as SLE and which may lead to false-positive CDC-based crossmatches do not necessarily belong to the IgM isotype class but represent lymphocytotoxic, that is, complement-fixing autoantibodies of the IgG-isotype [10]. Additionally some studies demonstrating the detrimental effect of HLA-specific alloantibodies of the IgM isotype have been published, thus clearly showing the benefit to detect and not to destroy them [17]. Unfortunately these alloantibodies of the IgM isotype are as well eliminated using DTT/DTE for CDC-based crossmatching but are detected using our modification of the AMS-ELISA with secondary anti-IgG/M/A antibodies. Taken together the AMS-ELISA is a suitable tool which methodically circumvents false positive crossmatch reactions in the presence of accompanying autoimmune diseases hence clearly improving the outcome for the patients.

## Figures and Tables

**Table 1a:** (a)

Serum sample	CDC-CM (#)			AMS-ELISA-CM	
	PBL	T-cell	B-cell	Class I	Class II
05/2010	1	1	1	n.d.	n.d.
10/2010	2/4	1/2	4	neg.	neg.
01/2011	1	1	1	neg.	neg.

n.d.: not done due to the unequivocally negative outcome of the CDC-CM; neg.: negative; pos.: positive; #: NIH score system of the standard CDC-based crossmatch as a percent of positive/dead (red coloured) cells (%): 1: <10% (negative), 2: 10–20% (doubtfully positive), 4: 20–50% (weakly positive), 6: 50–80% (positive), 8: 80–100% (strongly positive).

**Table 1b:** (b)

Serum sample	Screening ELISA		Luminex analysis	
	Class I (Quik Screen)	Class II (B-Screen)	Class I	Class II
05/2010	neg.	neg.	n.d.	n.d.
07/2010	neg.	neg.	n.d.	n.d.
10/2010	neg.	neg.	neg.	neg.
01/2011	neg.	neg.	neg.	neg.

n.d.: not done; neg.: negative; the Luminex analysis was not performed using the samples taken in May 2010 and July 2010 due to the unequivocally negative result of the class I and II screening ELISA but was additionally used to analyse the sera taken in October 2010 and January 2011.

**Table 2a:** (a)

Serum sample	CDC-CM (#)			AMS-ELISA-CM	
	PBL	T-cell	B-cell	Class I	Class II
10/2009	6	6	8	neg.	neg.
10/2010	6/8	6	8	neg.	neg.

neg.: negative; pos.: positive; #: NIH score system of the standard CDC-based crossmatch as a percent of positive/dead (red coloured) cells (%) as shown in Table 1(a).

**Table 2b:** (b)

Serum sample	Screening ELISA/DynaChip (Ø)		CDC cell tray (PBL)
	Class I	Class II	Class I / II
07/2009	neg. (Ø)	neg. (Ø)	n.d.
10/2009	neg. (Ø)	neg. (Ø)	pos. (68% PRA)
01/2010	neg.	neg.	n.d.
04/2010	neg.	neg.	n.d.
07/2010	neg.	neg.	n.d.
10/2010	neg. (Ø)	neg. (Ø)	pos. (61% PRA)

neg.: negative; pos.: positive; n.d.: not done; the CDC-based cell tray analysis was performed during the autumnal screening (i.e., once a year in accordance with the guidelines of the European Federation of Immunogenetics (EFI)) using the samples taken in October 2009 and October 2010; Ø: additionally performed DynaChip analyses in all cases exhibiting negative results in accordance with the screening ELISA.

## Abbreviations

AMS: Antibody monitoring system
CDC: Complement-dependent cytotoxicity
CM: Crossmatch
DTE: Dithioerythritol
DTT: Dithiothreitol
DSA: Donor-specific antibodies
ELISA: Enzyme-linked immunosorbent assay
FACS-CM: Flow cytometric crossmatch
HLA: Human leukocyte antigen
mAb: Monoclonal antibody
SLE: Systemic Lupus Erythematosus
PRA: Panel reactive antibodies.
